# Osteochondritis Dissecans (OCD)-Derived Chondrocytes Display Increased Senescence, Oxidative Stress, Chaperone-Mediated Autophagy and, in Co-Culture with Adipose-Derived Stem Cells (ASCs), Enhanced Expression of MMP-13

**DOI:** 10.3390/jcm8030328

**Published:** 2019-03-08

**Authors:** Katarzyna Kornicka, Mohamad Al Naem, Michael Röcken, Marta Zmiertka, Krzysztof Marycz

**Affiliations:** 1 International Institute of Translational Medicine, Jesionowa, 11, Malin, 55-114 Wisznia Mała, Poland; kornicka.katarzyna@gmail.com (K.K.); zmiertka.marta@gmail.com (M.Z.); 2 Department of Experimental Biology, Wroclaw University of Environmental and Life Sciences, 50-375 Wroclaw, Poland; 3 Faculty of Veterinary Medicine, Equine Clinic-Equine Surgery, Justus-Liebig-University, 35392 Gießen, Germany; mohamad.al-naem@vetmed.uni-giessen.de (M.A.N.); Michael.Roecken@vetmed.uni-giessen.de (M.R.)

**Keywords:** chondrocytes, osteochondritis dissecans, horse, apoptosis, cartilage

## Abstract

Osteochondritis dissecans (OCD) in equids, especially in sport horses, has become a growing issue as it contributes to the occurrence of lameness. Thus the aim of this study was to investigate the cytophysiological properties of OCD chondrocytes including expression of chondrogenic genes, apoptosis, mitochondria dynamics and autophagy. Horse chondrocytes were isolated from healthy (HE) and OCD cartilages. Properties of cells were evaluated using multiple assays e.g., polymerase chain reaction (PCR), immunofluorescence, Western blot. OCD chondrocytes were characterized by increased apoptosis and senescence. Expression of chondrogenic genes (vimentin, aggrecan) was decreased while mRNA levels of matrix metalloproteinase 13 significantly upregulated in comparison to HE cells. Moreover, OCD cells displayed increased mitochondrial fusion while fission events were diminished. Interestingly, chaperone mediated autophagy was triggered in those cells and it predominated over macroautophagy. Furthermore, co-culture of LPS-treated chondrocytes with adipose-derived stem cells (ASC) decreased p62/sequestosome 1 (SQSTM) and increases MMP-13 expression in OCD cells. Our results suggest that OCD affected horse chondrocytes are characterized by senescent phenotype due to endoplasmic reticulum stress and mitochondria dynamics deterioration. Expression of chondrogenic markers is decreased in those cells while expression of chaperone mediated autophagy (CMA)-related genes increased. Increased malfunctioning of cells leads to loss of their functionality and capacity to maintain tissue homeostasis.

## 1. Introduction

Osteochondrosis (OC) is a disease of the cartilage which negatively affects joint homeostasis. OC is found not only in humans but also in other mammals like dogs and horses. It is considered a serious condition which contributes to reduced mobility, poor performance and early retirement of sport horses causing adversefinancial consequences. OC is usually accompanied by osteochondritis dissecans (OCD), which occurs due to deterioration of endochondral ossification in the epiphyseal or metaphyseal growth plate of horses. Thickened cartilage in its basal layers is characterized by deterioration of cellular differentiation which leads to necrosis, retention and development of fissures. Due to that state, cartilage separates and can become free-floating fragments in the joint cavity. In consequence, severe joint inflammation may lead to the development of osteoarthritis [1]. Approximately 50% of OCD-affected horses suffer from bilateral lesions, although one of the lesions is more severe. Due to the high costs of surgical intervention and poor prognosis in the treatment of large lesions, the economic consequences of OCD are extensive [2].

The study of OCD in horses is especially important since as a large animal model, the horse more accurately recapitulates OCD in humans than other animals. It was shown that both cartilage stiffness and joint volume reflects characteristics of human cartilage and its loading forces are comparable [3,4]. Moreover, the application of arthroscopy allows for diagnosis and creation of lesions. Further benefits include repeated synovial fluid sampling and controlled, athletic exercise [5,6].

Chondrocytes are resident cells of articular cartilage responsible for regulation of extracellular matrix (ECM) composition, arrangement and mechanical resilience. However, during development of OCD, healthy cells turn into different phenotypes to finally undergo apoptosis. Dysfunctional chondrocytes produce an increased amount of matrix metalloproteinases (MMP) while reducing secretion of ECM proteins [7,8]. Although macroscopic and microscopic parameters of OCD in horses and humans have been well described, no data exists regarding molecular complications in OCD chondrocytes in horses. It was shown that equine OCD-derived chondrocytes were characterized by significantly increased expression of collagen I, II, III, X, matrix metallopeptidase 13 (MMP-13), Sox-9 and significantly decreased expression of TIMP metallopeptidase inhibitor 2 and 3 than chondrocytes isolated from healthy cartilage [9].

One of the self-protective mechanism in chondrocytes is autophagy, which allows for removal of dysfunctional organelles and proteins by their delivery to lysosomes. Beclin1 and microtubule-associated protein 1A/1B-light chain 3 (LC3) were found to be a major regulator of autophagic pathways in mammals allowing for autophagosome formation. Autophagy is a constitutively active and protective process allowing for the maintance of homeostasis in healthy chondrocytes. It was shown, however, in osteoarthritis OA cartilage that Beclin1 and LC3 expression is reduced leading to increased occurrence of apoptosis [10]. It was also proved that autophagy is increased in the early stages of OA while it decreases during later stages [11]. However, no data exist about chaperone-mediated autophagy (CMA) which selectively delivers proteins directly into lysosomes with the involvement of a degradation tag, in healthy OA or OCD chondrocytes.

Apoptosis plays a critical role in maintaining the homeostasis of various tissues including cartilage. Previous studies have estimated that the rate of apoptotic chondrocytes in OA cartilage varies between 1%–20% [12,13]. The term “chondropotosis” relates to the characteristic type of cell death observed in chondrocytes during OA which slightly differs from classical apoptosis. Chondrocytes undergoing chondroptosis display the presence of autophagic vacuoles, enlarged endoplasmic reticulum (ER) and primary lysosomes and increased Golgi mass [14]. Both, chondrocyte apoptosis and the loss of extracellular matrix form a continuous cycle, in which they aggravate progression of each other. Still, the deep understanding of chondroptosis and its contribution to disease development is not well understood and contradictory reports regarding this process exist [15].

Recently, the role of mitochondria in pathogenesis of cartilage and joint diseases, has become the subject of renewed interest. Mitochondria are now a focus of biomedical research due to their role in development and the pathogenesis of age-related disorders. It was shown that mitochondrial dysfunction is correlated with increased levels on reactive oxygen species which leads to inadequate synthesis of ECM proteins, inflammation pathologic cartilage matrix calcification and increased chondrocyte apoptosis [16]. OA chondrocyte mitochondria are characterized by decreased activity of complexes I, II, III and lower adenosine triphosphate (ATP) synthesis rate. The compensatory mechanism which enable to maintain energy homeostasis is enhanced through mitochondria biogenesis [17]. Furthermore, mitochondrial dynamics which involves processes called fission and fusion is also essential for regulating the metabolic functions of chondrocytes. For example it was shown that mitochondrial fission 1 protein (Fis1) is significantly down-regulated in human OA chondrocytes compared to healthy cells and it induced accumulation and inhibition of lysosomes [18]. Deteriorated, dysfunctional organelles can be removed from cells via a specific form of autophagy called mitophagy. It was shown that activation of autophagy protects chondrocytes against mitochondrial dysfunction most likely by removal of damaged organelles and thus preventing oxidative stress [10]. In order to remove dysfunctional mitochondria, Parkin (E3 ubiquitin ligase) is selectively recruited to their membrane allowing them to be engulfed by autophagosomes. It was shown that OA chondrocytes are characterized by increased Parkin expression which protects them against reactive oxygen species (ROS) [19]. However, no data regarding mitochondria dynamics in OCD chondrocytes exists.

Another critical factor for chondrocyte deterioration is ER stress [20] Accumulation of misfolded proteins in the ER lumen triggers the unfolded protein response (UPR), which activates C/EBP homologous protein (CHOP) and caspases inducing chondrocyte death [21,22]. Recent data showed that ER stress inducer, tunicamycin, triggered CHOP and MMP-13 expression while decreasing collagen type II and aggrecan mRNA levels [23]. On the other hand, it was shown that targeting ER stress may become effective in the OA treatment [24]. Involvement of ER stress in the pathogenesis of OA [25] has been well studied, however the cross-talk of ER stress and OCD development is still missing.

The aim of this study was to answer the investigate the cytophysiological properties of equine OCD chondrocytes at molecular level. For that reason we investigated viability, chondrogenic and hypertrophic genes expression, senescence, apoptosis, ER stress, mitochondrial dynamics and autophagy in OCD-derived chondrocytes. It is absolutely necessary to establish the molecular properties of those cells while taking into consideration the testing of new drugs against OCD and their translation into human cartilage regeneration. As it has been performed in-depth in humans, the pathophysiological events in equine chondrocytes are less clear.

## 2. Experimental Section

All reagents used in this experiment were purchased from Sigma-Aldrich (Poland), unless indicated otherwise.

### 2.1. Animals and Collection of Cartilage Samples

The cartilage samples were removed during arthroscopic surgery as a part of the standard curettage of the subchondral bone after removing the OC-fragment, and further medical waste were collected for that study, explaining why agreement of the local ethical committee was not necessary. The cartilage was obtained from 7 horses, aged between 9–11 years, of both sexes, of body weight between 570–630 kg, and fed with commercial feed. The horses were used in a jumping discipline of low intensity. In all horses radiological evidence of fragmentation were visible. Arthroscopy was undertaken on the basis of one or more of the following features: synovial effusion, lameness localised to the affected joint. Before arthroscopic operation, the joint were swollen and inflamed. The chondrocytes were isolated from cartilages pieces that floated in the fetlock joint. Immediately after removal, fragments were inserted into the transport medium and delivered to the Experimental Biology Department at Wroclaw Environmental and Life Science University. Healthy cartilage was derived post mortem from 6 horses, aged between 9–12 years of both sexes, body weight between 550–645 kg, fed with commercial feed. Cartilage were harvest from the fetlock joint.

### 2.2. Adipose-Derived Stem Cells (ASC) and Chondrocytes’ Isolation and Culture

Cartilage from healthy and OCD horses were placed in a Hank’s balanced salt solution (HBSS) and washed vigorously. Next, the specimens were minced into small pieces and transferred to 1 mg/mL clostridial collagenase at 37 °C for 8 h. Then, the solution was filtered using 70 μm strainers following centrifugation at 1200 rpm for 8 min. After discarding the supernatant, the cell pellet was washed three times with HBSS. Cells were suspended in Dulbecco′s modified Eagle′s medium (DMEM) Hams-F12 with 10% fetal bovine serum (FBS), 1% penicillin/streptomycin/amphotericin (P/S/A) and cultured at 37 °C, 95% air with 5% CO2.

Adipose-derived stem cells (ASC) were isolated from healthy horses in accordance to procedure described previously [26]. Ethics approval for the adipose tissue harvesting was obtained from Local Research Ethics Committee in Wroclaw (approval no. 84/2018). Briefly, subcutaneous adipose tissue (from the tail base of horses) was harvest and placed in HBSS supplemented with 1% P/S/A solution. Specimens were cut into small pieces with surgical scissors and transferred to collagenase type I (1 mg/mL) following incubation for 40 min at 37 °C. Then samples were centrifuged at 1200× *g* for 10 min at 23 °C and re-suspended in culture media (DMEM) with low glucose supplemented with 10% of FBS and 1% P/S/A solution.

After reaching 80%–90% of confluence, both ASC and chondrocytes were passaged using a trypsin solution (TrypLE Express, Life Technologies). Media were changed every 2–3 days.

### 2.3. Evaluation of Viability and Proliferation Rate

In order to evaluate viability, chondrocytes were seeded onto 24-well plates at the density of 4 × 10^4^ cells per well. After 24 h, cells were subjected for analysis using resazurin-based assay (TOX-8) in accordance with the manufacturer′s protocol. Briefly, culture media were collected and substituted with medium containing 10% of resazurin dye. Following 2 h incubation in a CO_2_ incubator, 100 µ of medium from each well was transferred onto a 96-well plate. Absorbance was measured spectrophotometrically using a 96-well microplate reader (Epoch, BioTek, Winooski, VT, USA). Reduction of the dye was evaluated at a wavelength of 600 nm and a reference wavelength of 690 nm.

In order to investigate proliferation rate of cells, we performed the assay based on the BrdU incorporation into the newly synthesized DNA using the BrdU Cell Proliferation enzyme-linked immunosorbent assay (ELISA) Kit (Abcam, Cambridge, UK) following the manufacturer’s protocol. Briefly, cells were seeded onto a 96-well plate at a concentration of 1 × 10^4^ of cells per well. After the attachment of cells, BrdU reagent was added in a volume of 20 μL. Then cells were incubated for 24 h at 37 °C in CO_2_ incubator. Next, cells were fixed and DNA was denaturated with supplied reagents. Incorporated BrdU was detected with anti-BrdU monoclonal antibody and horseradish peroxidase-conjugated goat anti-mouse IgG secondary antibody. Reaction was developed using chromogenic substrate tetra-methylbenzidine (TMB). Then, absorbance at 450 nm wavelength was measured.

### 2.4. Evaluation of Cellular Morphology

The morphology of chondrocytes was evaluated with a scanning electron microscope (Zeiss EVO LS15, Oberkochen, Germany). Prior experimental cells were washed with HBSS and fixed with 4% paraformaldehyde (PFA) for 45 min in room temperature. Next, cells were dehydrated in a graded ethanol series (concentration from 50% to 100%) and sputtered with gold (ScanCoat 6, Edwards, Oxford, UK), transferred to microscope chamber and observed using a SE1 detector, at 10 kV of filament′s tension.

Prior LAMP-2 visualization, cell membranes were permeabilized with 0.5% Triton X-100 for 20 min at room temperature while unspecific binding sites were blocked with blocking buffer (10% Goat Serum, 0.2% Tween-20 in HBSS) for 45 min. Cells were then incubated overnight at 4 °C with primary antibodies against LAMP2 (Abcam) diluted 1:500 in HBSS containing 10% Goat Serum. Cells were then washed again and incubated for 1 h with goat anti-mouse secondary antibodies conjugated with atto-488 (dilution 1:1000, Abcam), avoiding direct light. Specimens were mounted in ProLongGold (Life Technologies, Carlsbad, CA, USA) and were observed using confocal microscope (Olympus Fluoview FV1200, Shinjuku, Tokio, Japan).

In order to visualize f-actin, cells were fixed in 4% PFA and permeabilized with 0.2% Tween 20 in HBSS for 15 min. Then specimens were incubated with phalloidin Atto594 in HBSS (1:800) in the dark for 45 min. Cell nuclei were counterstained with 4′,6-diamidino-2-phenylindole (DAPI). Photographs were taken using a confocal microscope, Olympus Fluoview FV1200.

Proteoglycans were stained using Safranin O. Briefly, cells were fixed with 4% PFA and stained with Safranin O solution for 30 min at room temperature. To visualize senescence-associated β-galactosidase, we applied Senescence Cells Histochemical Staining Kit following the manufacturer′s protocol. Photographs were taken using an inverted bright field and epifluorescent microscope (Zeiss, Axio Observer A.1, Oberkochen, Germany).

In order to visualize the mitochondrial net, chondrocytes were incubated in medium containing MitoRed dye (1:1000) for 30 min. After washing with HBSS, cells were fixed in 4% PFA and nuclei were counterstained with DAPI. Mitochondria depolarization was observed using JC-1 fluorescent probe (Thermo Scientific, Waltham, MA, USA) staining. 5 μM JC-1 was added to cells following 30 min incubation at 37 °C. Next, cells were washed with HBSS and fixed with 4% PFA. Additionally, viable and dead cells were visualized using the Cellstain Double Staining Kit following manufacturer’ instruction. Briefly, live cells were stained with Calcein A.M while apoptotic with propidium iodide. Specimens were observed under epi-fluorescent microscope (Zeiss, Axio Observer A.1, Oberkochen, Germany).

### 2.5. Measurement of Superoxide Dismutase (SOD) Activity and Nitric Oxide (NO) Concentration

In order to perform the assays, culture media were collected after 24 h of culture. Superoxide dismutase (SOD) activity was evaluated using SOD assay kit. It determines SOD activity in an indirect assay method based on xanthine oxidase and a novel color reagent. Water-soluble formazan dye undergoes reduction with a superoxide anion and the rate of the reduction is linearly related to the xanthine oxidase (XO) activity, and is inhibited by SOD. Thus SOD activity is calculated as inhibition rate in%. NO levels were measured using commercially available Griess reagent kit (Life Technologies, Carlsbad, CA, USA)-colorimetric assay for nitrites, and nitrates that have been reduced to nitrites. Procedures were carried out following the manufacturer’s recommendations.

### 2.6. Chondrocytes-ASC Co-Culture

ASC was cultured in a transwell insert at a density of 70 × 10^3^ cells per well while chondrocytes were propagated on the bottom at the density of 50 × 10^4^ cells per well. Both cells population were cultured in DMEM Hams-F12 with 10% FBS and 1% of PSA. Prior to co-culture, 1µg/mL of lipopolysaccharide (LPS) was added to treat the chondrocytes for 2 h. Then, ASC were added and indirectly co-cultured with chondrocytes for 24 h. After that, cells were subjected for reverse transcription polymerase chain reaction (RT-PCR) analysis.

### 2.7. Western Blotting

In order to perform Western blotting, cells were detached from culture dishes and homogenized in radioimmunoprecipitation assay buffer (RIPA) buffer supplemented with protease inhibitor cocktail. The lysates were centrifuged at 4 °C for 20 min (14,000g) and supernatants were transferred to new tubes. Thirty micrograms of protein were used for each sample. SDS-PAGE was performed at 100 V for 90 min. Proteins were transferred onto nitrocellulose membrane (Bio-Rad) using a transfer apparatus at 100 V for 1 h at 4 °C. Next, membranes were washed with Tris/NaCl/Tween buffer (TBST) and blocked overnight at 4 °C with 5% non-fat milk in TBST. Finally membranes were incubated overnight with primary antibody for mitochondrial fission factor MFF (Biorbyt orb325479) at a dilution of 1:500. Following washing, solution of appropriate secondary antibody conjugated with horseradish peroxidase (HRP) was applied. After 2 h incubation, the membrane was washed again with TBST and incubated with Luminata Forte substrate (Merck) and visualized with ChemidocMP (Biorad).

### 2.8. Real-Time Reverse Transcription Polymerase Chain Reaction (RT-PCR)

Total RNA was isolated from cells according to a method described previously by Chomczynski and Sacchi [27] and RT-PCR was performed as described previously [28]. RNA quality and quantity were determined with spectrophotometry (Epoch, BioTek, Winooski, VT, USA). 150 ng of total RNA was used for genomic DNA digestion and cDNA synthesis. Digestion of genomic DNA was performed using DNase I, RNAase-free (Life Technologies, Carlsbad, CA, USA) while complementary DNA (cDNA) synthesis was performed using the RevertAid RT Reverse Transcription Kit (Life Technologies, Carlsbad, CA, USA). The qRT-PCR reactions were performed using a SensiFAST SYBR Green Kit (Bioline, London, UK). Primer concentration equalled 0.5 μM. Sequences of the primers used in reactions are listed in Table 1. All qRT-PCR reactions were conducted with the CFX Connect™ Real-Time PCR Detection System (Bio-Rad, Hercules, CA, USA). Relative expression of genes was evaluated in relation to the GAPDH as a housekeeping gene using the ΔΔCt method.

### 2.9. Statistics

The results were evaluated based on measurements obtained in subsequent repetitions. All experiments were performed at least in triplicate. Assessment of the normality of data was performed with the Shapiro–Wilk test. Differences between groups were determined using the non-parametric t-test. Statistical analysis was performed with GraphPad Prism 5 software (La Jolla, San Diego, CA, USA). Differences were considered statistically significant for *p* < 0.05.

## 3. Results

### 3.1. Metabolic Activity and Morphology

**Figure 1:**
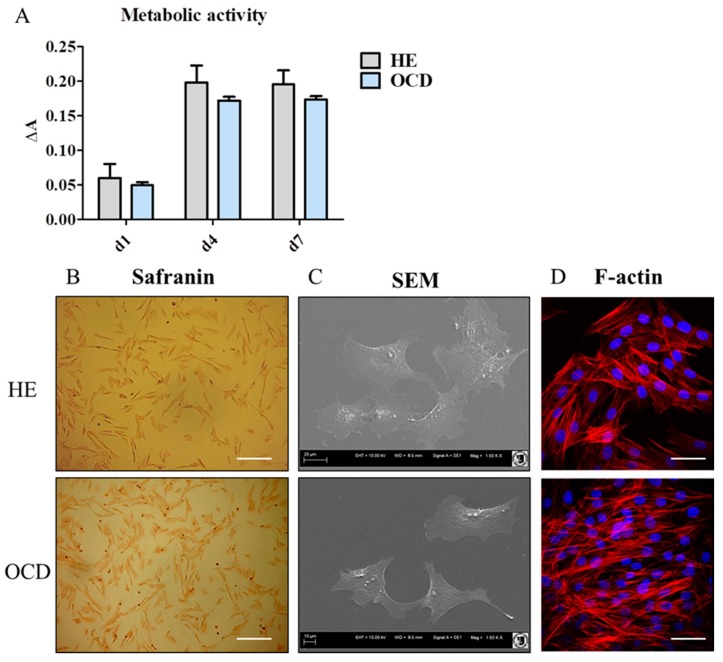
Metabolic activity and morphology of healthy chondrocytes (HE) and osteochondritis dissecans (OCD) chondrocytes. Metabolic activity was assessed using Alamar Blue assay after day 1 (d1), 2 (d2) and 7 (d7) of culture (**A**). Chondrocytes were stained with safranin and observed under phase contrast microscope (**B**). Furthermore, cell were observed in scanning electron microscopy (SEM) (**C**). F-actin was visualized using phallodin and captured with confocal microscope (**D**). Scale bars: brightfield-250 μm, confocal: 50 μm. Results expressed as mean ± standard deviation (S.D.).

Metabolic activity was established with TOX-8 reagent [Figure 1A]. Interestingly, there were no differences in metabolic activity of cells at day 1, 4 and 7 of culture. Safranin staining confirmed the accumulation of proteoglycans in both, healthy (HE) and OCD cells [Figure 1B]. Scanning electron microscopy (SEM) [Figure 1C] and f-actin [Figure 1D] revealed that HE chondrocytes were characterized by polygonal morphology while OCD cells displayed more fibroblastic shape.

### 3.2. Expression of Chondrogenic and Hypertrophic Genes

**Figure 2:**
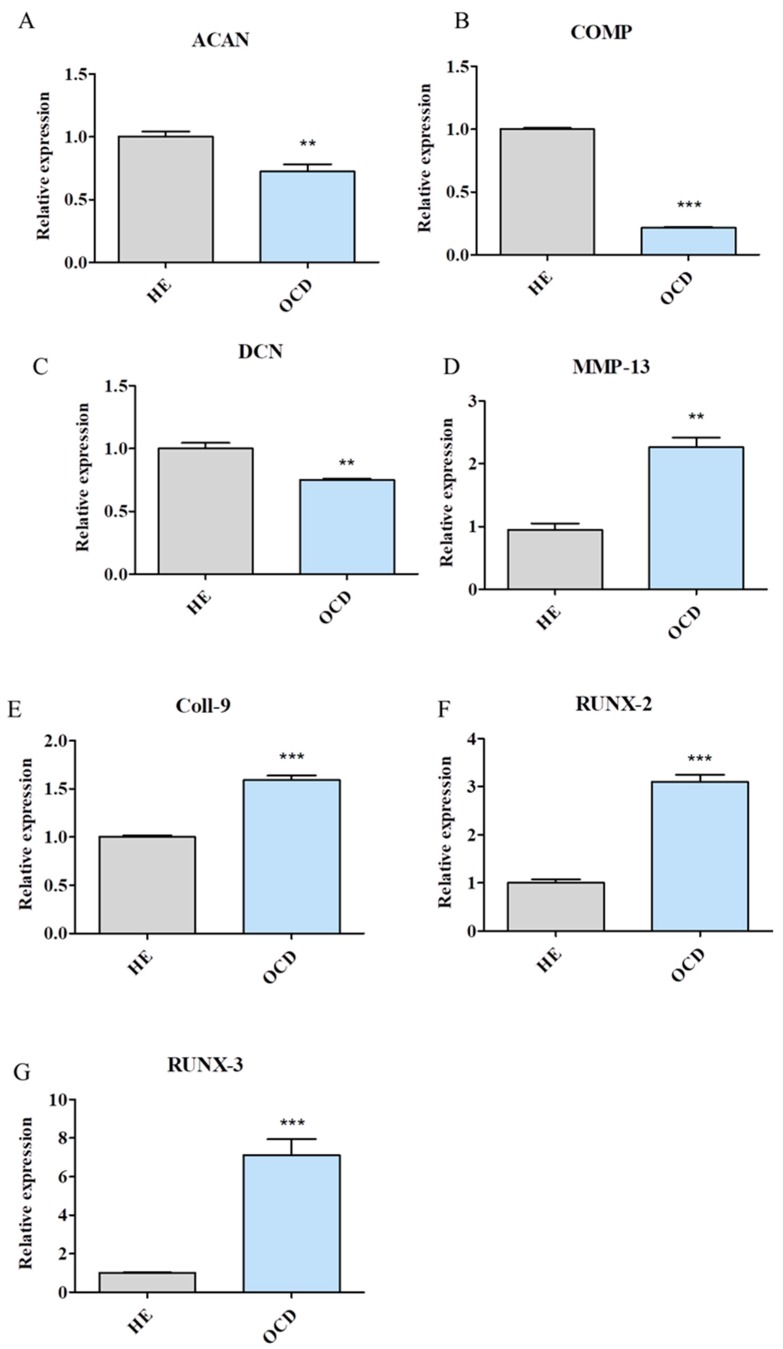
Using RT-PCR expression of key chondrogenic and hypertrophic genes was investigated. mRNA levels of following genes were analysed: ACAN (**A**), COMP (**B**), DCN (**C**), MMP-13 (**D**), Coll-9 (**E**), RUNX-2 (**F**) and RUNX-3 (**G**). OCD chondrocytes were characterized by hypertrophic phenotype and decreased expression of ECM genes. Results expressed as mean ± S.D. ** *p* < 0.01, *** *p* < 0.001.

Expression of aggrecan (ACAN), which maintains chondrocytes phenotype by interactions with glycosaminoglycans was significantly decreased in OCD cells [Figure 2A]. Similarly, expression of ECM proteins COMP (cartilage oligomeric matrix protein) [Figure 2B] and DCN (decorin) [Figure 2C] was also reduced in those cells. On the other hand, mRNA levels of MMP-13 (matrix metallopeptidase) which promotes the degradation of ACAN and collagen type II was significantly upregulated in OCD chondrocytes [Figure 2D]. Analogous phenomenon was noted in expression of other hypertrophic genes e.g., Coll-9 (collagen IX) [Figure 2E], RUNX-2 (runt-related transcription factor 2) [Figure 2F] and RUNX-3 (runt-related transcription factor 3) [Figure 2G].

### 3.3. Apoptosis and Senescence

**Figure 3:**
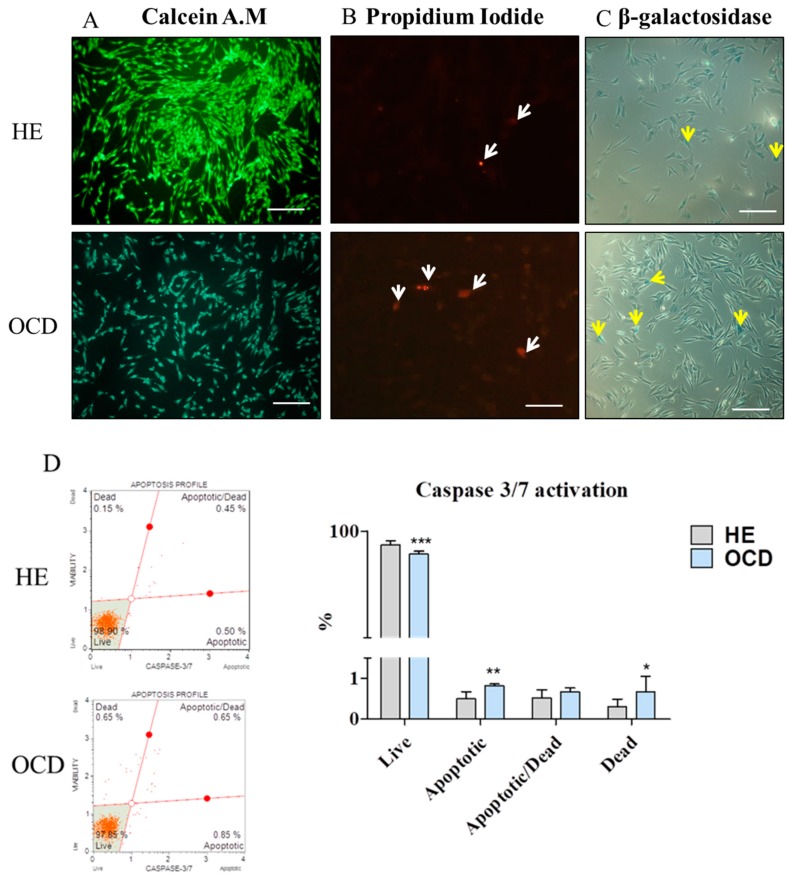

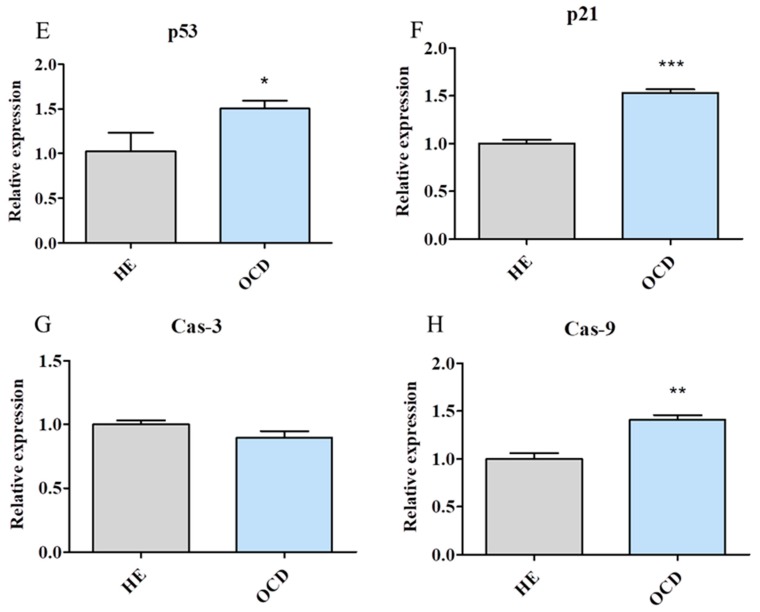
Evaluation of senescence and apoptosis. Live chondrocytes were stained with Calcein A.M (**A**) while nuclei of dead cells with propidium iodide (indicated with white arrows, **B**). Cells were observed under epi-fluorescent microscope. Moreover accumulation of senescence-associated β-galactosidase was visualized (indicated with yellow arrows, **C**) and imaged using wide field inverted microscope. Apoptotic status of chondrocytes based on caspase-3/7 activation, cellular plasma membrane permeabilization and death was established using the MUSE Cell Analyzer (**D**). In addition expression of apoptotic-related genes eg. p53 (**E**), p21 (**F**), cas-3 (**G**) and cas-9 (**H**) was evaluated using RT-PCR. Scale bars: epifluorescent: A-250 μm, B-100 μm, brightfield-150 μm. Results expressed as mean ± S.D. * *p* < 0.05, ** *p* < 0.01, *** *p* < 0.001.

HE chondrocytes were characterized by 80% confluence while OCD displayed slight loss of calcein fluorescence intensity [Figure 3A]. What is more those cells were characterized by increased apoptosis, as in indicated by propidium iodide staining [Figure 3B] and senescence- increased number of senescent-positive (blue) cells [Figure 3C]. Using the MUSE Cell Analyzer and Caspase 3/7 activation kit, percentage of live, dead, apoptotic, apoptotic/dead cells in cultures was assessed [Figure 3D]. Results indicated that the number of live cells was decreased while the number of apoptotic and dead cells increased in OCD groups which corresponds with calcein and propidium iodide staining. Expression of pro-apoptotic genes p53 [Figure 3E] and p21 [Figure 3F] was significantly increased in OCD chondrocytes. Interestingly, no differences were found in the expression of cas-3 (caspase 3) [Figure 3G] while mRNA levels of cas-9 (caspase 9) [Figure 3H] was increased in OA cells.

### 3.4. Expression of Endoplasmic Reticulum (ER) Stress-Related Genes

**Figure 4:**
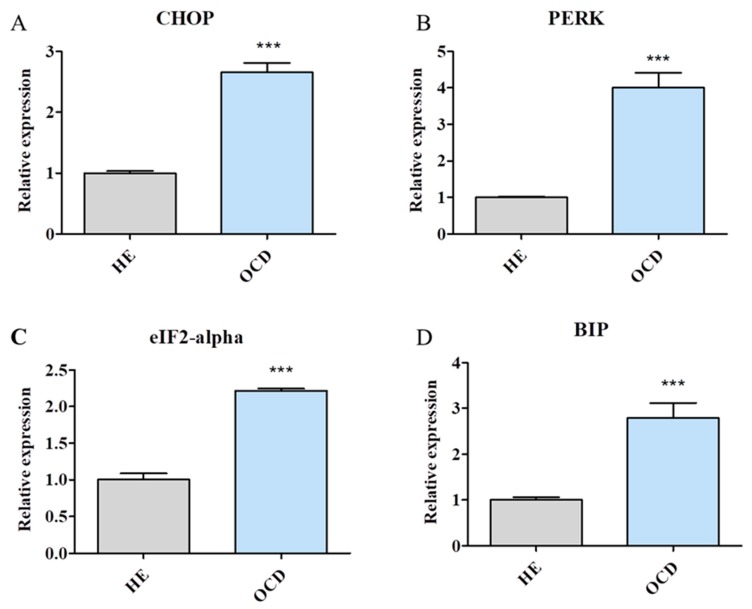
Expression of endoplasmic reticulum (ER)-stress related genes. In order to investigate whether OCD triggers ER stress in chondrocytes expression of CHOP (**A**), PERK (**B**), aIF2-aplha (**C**) and BIP (**D**) was investigated with RT-PCR. Results expressed as mean ± S.D. *** *p* < 0.001.

Expression of genes which participate in ER stress response including CHOP [Figure 4A], PERK [Figure 4B], eIF2-alpha [Figure 4C] and BIP [Figure 4D] was tested. mRNA levels of those genes were significantly upregulated in OCD chondrocytes in comparison to normal chondrocytes.

### 3.5. Assessment of Mitochondria Condition

**Figure 5:**
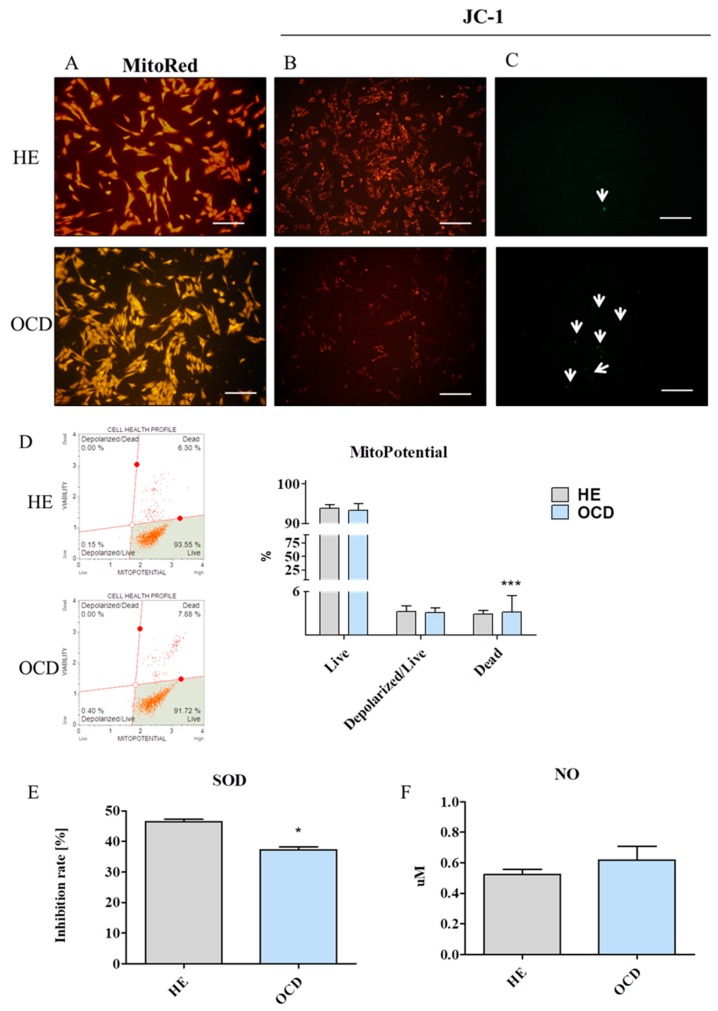
Evaluation of mitochondria condition in equine HE and OCD chondrocytes. Mitochondria of chondrocytes were stained using MitoRed dye and observed under epifluorescent microscope (**A**). Furthermore, to investigate mitochondria condition, cells were stained with JC-1. Mitochondrial depolarization is indicated by a decrease in the red and increase in the green fluorescence. Polarized mitochondria are marked by orange-red fluorescence (**B**) while depolarization is indicated with green fluorescence (**C**). Quantitative data regarding the mitochondria condition was obtained using MitoPotential assay and MUSE Cell Analyzer (**D**). In order to evaluate antioxidative status of chondrocytes activity of superoxide dismutase (SOD) was established (**E**). Furthermore, amount of NO in culture media was evaluated (F). Scale bars: epifluorescent −250 μm. Results expressed as mean ± S.D. * *p* < 0.05, *** *p* < 0.001.

Mitochondria of OCD cells were characterized by decreased fluorescence intensity which may indicate diminished metabolic activity of those organelles [Figure 5A]. Furthermore, to investigate the mitochondrial membrane potential (MMP), cells were stained with JC-1 dye and observed under epifluorescent microscope. Number of mitochondria stained with red/orange was downregulated in OCD chondrocytes in comparison to healthy cells [Figure 5B]. On the other hand the number of mitochondria emitting green fluorescence (with decreased MMP) was greater in OCD cells than in HE [Figure 5C]. MMP was further evaluated using the MUSE Cell Analyser [Figure 5D]. The data obtained revealed that increased number of dead cells in OCD group. In order to evaluate antioxidative defence of isolated cells, activity of SOD was tested [Figure 5E]. OCD chondrocytes displayed decreased SOD activity in comparison to healthy cells. Amount of extracellular NO was comparable between groups [Figure 5F].

### 3.6. Mitochondrial Dynamics and Autophagy

**Figure 6:**
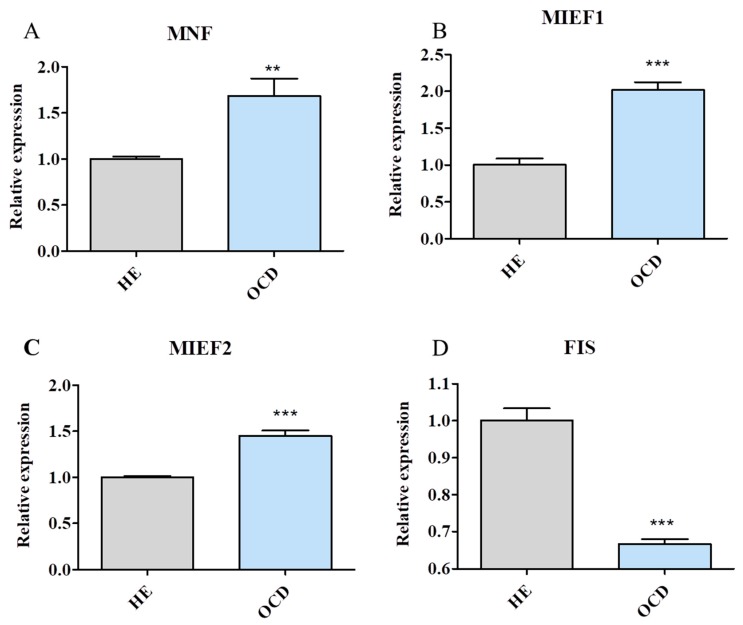

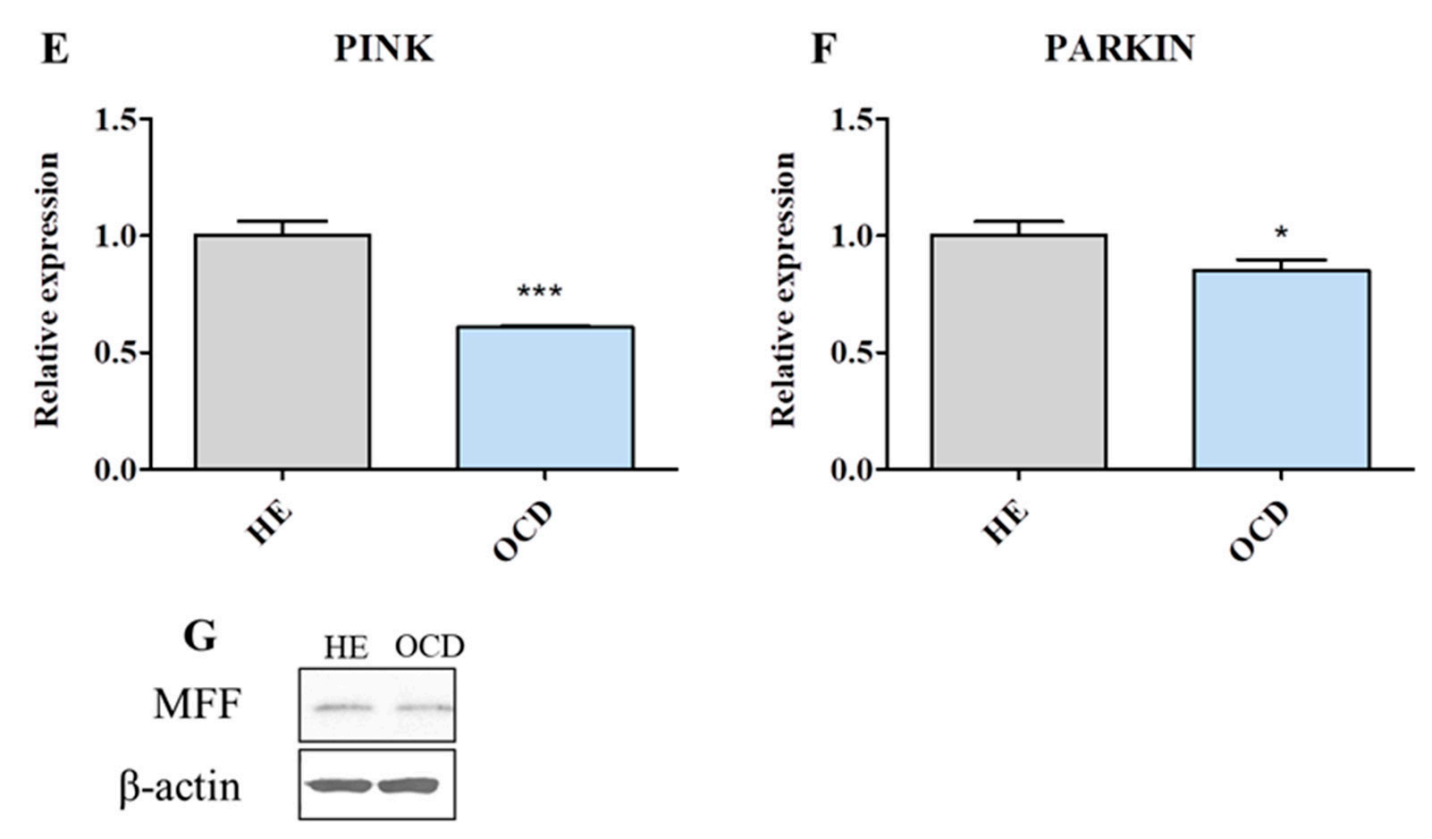
Assessment of mitochondrial dynamics and mitophagy. HE and OCD chondrocytes were analysed by RT-PCR for mRNA expression. Expression of MNF (**A**), MIEF1 (**B**) and MIEF2 (**C**) was established in order to compare intensity of mitochondrial fusion in investigated groups. To analyse mitochondrial fission, FIS expression was investigated (**D**). Moreover, expression of mitophagy markers: PINK (**E**) and PARKIN (**F**) was analysed. Mitochondrial fission factor (MFF) protein level was established by Western blot (**G**). Results expressed as mean ± S.D. * *p* < 0.05, ** *p* < 0.01, *** *p* < 0.001.

Expression of genes involved in the process of mitochondrial fusion, including MNF (mitofusin 1) [Figure 6A, MIEF-1 (mitochondrial elongation factor 1) [Figure 6B] and MIEF2 (mitochondrial elongation factor 2) [Figure 6C] was investigated. Interestingly expression of those genes was significantly upregulated in OCD chondrocytes. Contrary, expression of protein related to mitochondrial fission, FIS [Figure 6D] was significantly downregulated in those cells. Furthermore, expression of genes involved in the progression of mitophagy, PINK (PTEN-induced kinase 1) [Figure 6E] and PARKIN (parkin ligase) [Figure 6F] was downregulated in those cells. Amount of MFF protein was investigated with western blot [Figure 6G] however no significant difference between HE and OCD band thickness was observed.

### 3.7. Evaluation of Autophagy

**Figure 7:**
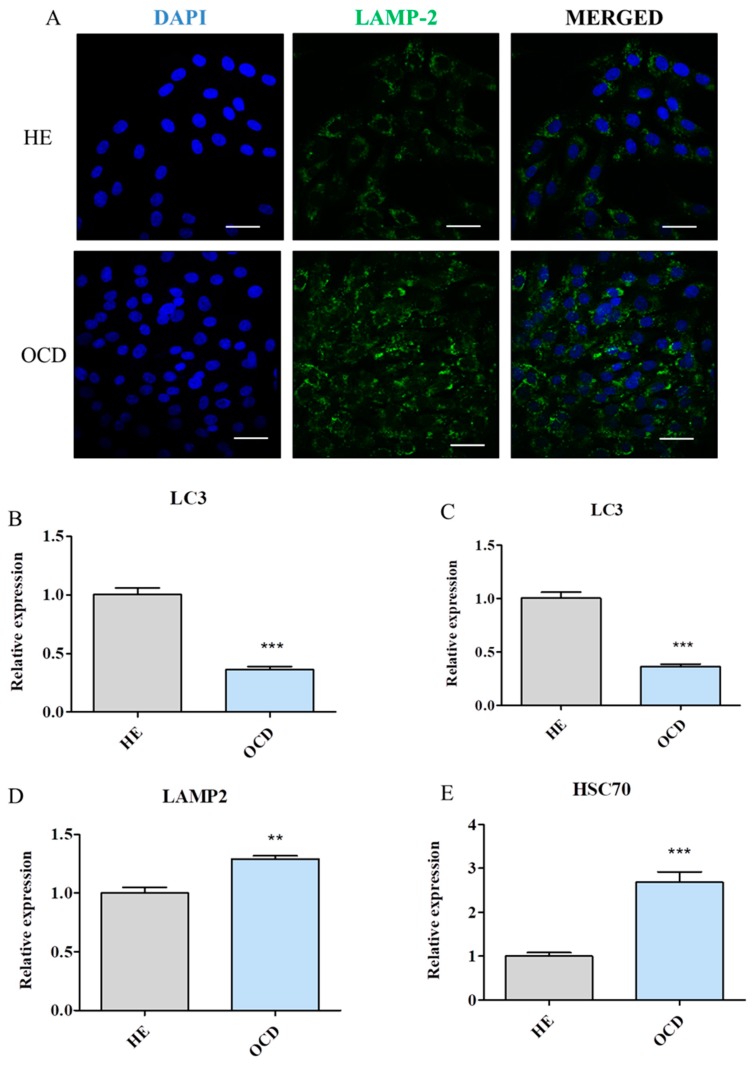
Evaluation of autophagy in HE and OCD chondrocytes. Using immunofluorescence staining, LAMP-2 accumulation in cells was visualized using confocal microscope (**A**) Using RT-PCR expression of LC3 (**B**), BECLIN (**C**), LAMP-2 (**D**) and HSC70 (**E**) was established. Scale bars: confocal: 60 μm. Results expressed as mean ± S.D. ** *p* < 0.01, *** *p* < 0.001.

Immunofluorescence staining revealed increased accumulation of LAMP-2 in OCD chondrocytes [Figure 7A]. Furthermore, expression of autophagy related genes was investigated with RT-PCR. Interestingly LC-3 mRNA levels were diminished in OCD cells [Figure 7B] while no differences were noted in BECLIN expression [Figure 7C]. What is more, expression of LAMP-2 [Figure 7D] and HSC70 [Figure 7E] was significantly upregulated in OCD chondrocytes which indicates that not macroautophagy but CMA predominates in those cells.

### 3.8. Chondrocytes-ASC Co-Culture

**Figure 8:**
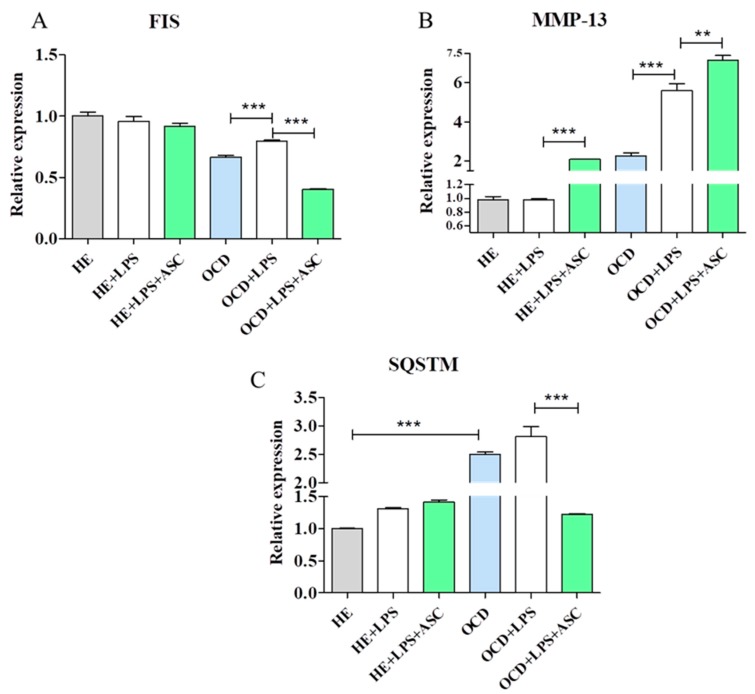
FIS, MMP-13 and SQSTM expression in HE and OCD chondrocytes. Cells were treated with 1 µg/mL LPS in monoculture or co-culture with ASC for 24 h. Then, cells were analysed by RT-PCR for FIS (**A**), MMP-13 (**B**) and SQSTM (**C**) expression. Results expressed as mean ± S.D. ** *p* < 0.01, *** *p* < 0.001.

The catabolism of chondrocytes was triggered with LPS treatment for 2 h. Then ASC were added to chondrocytes and co-cultured for 24 h. After that period cells were subjected for RT-PCR analysis. Interestingly, mitochondrial fission was diminished in OCD cells after co-culture with ASC as decreased expression of FIS was observed [Figure 8A]. What is more, ASC enhanced the expression of MMP-13 in OCD cells [Figure 8B]. Furthermore, during co-culture with ASC autophagy diminishes in OCD chondrocytes as decreased SQSTM expression was decreased in those cells [Figure 8C].

## 4. Discussion

In this study we have shown for the first time the comprehensive analysis of healthy and OCD chondrocytes isolated from horses. We have analysed apoptosis, expression of ECM proteins, oxidative stress, autophagy and mitochondrial dynamics in order to reveal the major causes of chondrocytes deterioration during OCD. It is especially important to unravel and compare the characteristics of chondrocytes from humans and equids as horse have become a model to study OA in humans. Pathogenesis of those two diseases may be distinct, although deterioration of cellular functionality seems to be comparable which indicates that chondrocytes respond for OA and OCD in similar manner. However, an in-depth analysis of the molecular characteristics of equine chondrocytes is currently missing. In our study we revealed that OCD chondrocytes share a set of common characteristics to previously studied OA tissue. Although some molecular changes are common for those cells, they consequences are distinct.

It was shown that both OA and OCD chondrocytes are characterized by increased senescence and degeneration [29,30]. Similarly, in our study we have observed increased expression of pro-apoptotic genes, increased number of apoptotic cells, and accumulation of senescence-associated β-galactosidase. It is worth noting that senescent chondrocytes lose their ability to replace their extracellular matrix. This suggests the hypothesis that senescence of cells within joint tissues may play a pathological role in the causation of OCD. In their study Xu et al. [31] injected either senescent or non-senescent cells into the knee joint area of mice and revealed that transplanting senescent cells caused pathological features suggestive of OA which highlights the importance of senescent cells in development of musculoskeletal disorders. In study performed by Kim et al. [32], it was shown that chondrocyte apoptosis from the OA horse’ cartilages were significantly higher than from healthy cartilages. Although the precise molecular mechanism of senescence in chondrocytes is still not elucidated, it is thought to be associated with vascular endothelial growth factor (VEGF), p53, p21 and p16 [33]. In our study we have observed increased expression of p21, p53, BAX and caspase-9 in OCD chondrocytes. Activation of p53 is triggered by DNA damage and telomere shortening in order to inhibit cell cycle progression. Up-regulation of 53 activates p21 which leads to cellular senescence. Dai et al. [34] revealed that stress-induced senescence of chondrocytes is accompanied by enhanced expression of both p21 and p53. It was showed that chondrocytes expressing p53 possesses similar to OA chondrocytes morphology and downregulation of p53 expression can prevent apoptosis and senescence in those cells [35]. On the other hand, increased expression of caspase-3 and caspase-9 was noted, however, in human OA chondrocytes [36]. In this study, we revealed that OCD chondrocytes, similar to OA cells, highly express pro-apoptotic genes. Increased apoptosis and senescence in OCD chondrocytes and the interplay between these processes may be crucial in the pathogenesis and progression of that disease. Similar results were obtained by Semevolos et al. [30] who observed increased apoptosis of osteochondral junction chondrocytes from OC horses. Other mechanisms of cell death may be failing in OCD, thus apoptosis is triggered in order to induce cell death and ossification. It is supported by the fact that the OCD hallmark feature is delayed ossification of epiphyseal cartilage which in consequence leads to pain and decreased mobility [30]. Thus it is tempting to speculate that apoptosis and senescence of chondrocytes are the major cause of OCD development and progression. That thesis is supported by the fact that thymosin-β4- protein involved in inhibition of actin polymerization and regulation of apoptosis is increased in experimentally-induced osteochondrosis [37]. It highlights the importance of chondrocyte apoptosis during OCD.

Previous studies have shown that apoptosis can be considered as a marker for chondrocytes hypertrophy [38]. Hypertrophic chondrocytes are characterized by the expression of specific proteins such as RUNX-2, type X collagen, MMP-13 and osteocalcin. Increased expression of MMP-13 in OCD cells correlates with multiple other reports that indicated on its upregulation during pathological changes of cartilage including OA [39,40]. Similar results were obtained by Laverty et al. [41], who showed MMP-13 elevated activity in equine OC cartilage. What is more, it was shown that MMP-13 is activated by RUNX-2 overexpression by mitogen-activated protein kinase (MAPK) pathways and direct regulation of MMP-13 gene transcription [42,43]. As RUNX-2 plays a pivotal role in chondrocytes differentiation and matrix degeneration it was shown to be overexpressed in both, OC and OA cartilage [44,45]. Recently, RUNX-3 has been identified as a new RUNX2 target, which cooperatively regulates hypertrophic differentiation of ATDC5 chondrocytes [46]. This study also demonstrated that similar to those in humans, equine OA chondrocytes are characterized by hypertrophic phenotype as increased expression of MMP-13, RUNX-2 and RUNX-3 was observed. On the other hand, the expression of collagen type II, COMP and DCN was augmented in those cells which supports those observed in human OA degeneration of ECM [47].

An alternative explanation of OCD chondrocytes senescence is based on the damage caused by oxidative stress. Oxidative stress occurs when levels of nitric oxide (NO) and ROS exceeds the antioxidant capacities of cells. Many studies have shown that NO and ROS may be directly involved in the pathogenesis of musculoskeletal disorders [48,49] but no data regarding its involvement in OCD exist. To clarify the role of oxidative stress and mitochondria in OCD chondrocytes we looked for the presence of oxidative damage in those cells. The results obtained clearly indicated for the first time that oxidative stress affects OCD horses’ chondrocytes contributing to increased apoptosis, ER stress and autophagy. Our findings are consistent with a large body of data showing that ROS are a major factor, however, in the development of OA [50,51]. We have shown that horse OCD chondrocytes are characterize by enhanced ROS accumulation and NO synthesis while SOD activity in those cells is significantly diminished. Thus, excessive ROS accumulation seems to be the mechanism underlaying chondrocyte deterioration in both, OA and OCD. Increased NO production from horse chondrocytes was indirectly evaluated by immunohistochemistry by Kim et al. who observed increased NO accumulation in cartilage from OA horses [32]. On the other hand, Fu et al. [52] have shown that decreased SOD activity was SIRT-3 dependent and that its expression was downregulated in human and mice OA cartilage. As a result of the overload with damaged and misfolded proteins in deteriorated chondrocytes, the activation of ER stress pathways occurs. Excessive ER stress leads to the activation of CHOP which triggers apoptosis. It was reported that ER stress-induced apoptosis and enhanced CHOP expression contributed to chondrocyte apoptosis along with OA progression [53]. This is in good agreement with our data as we observed increased expression of ER stress mediators such as CHOP, PERK and BIP in OCD horse chondrocytes. It corresponds with increased apoptosis in those cells as CHOP leads to decreased expression of Bcl-2 while enhancing the expression of BAX and caspases [54]. Moreover, similar to our results, it was shown that CHOP induces the expression of MMP-13 while decreasing the amount of collagen type II and aggrecan [55].

Numerous studies have shown that stress-triggered senescence is manifested by mitochondria damage and that mitochondria deterioration plays a key role in the production of ROS. Mitochondria serve as key organelles for energy production and protects cells from oxidative damage. However, the loss of mitochondria functionality has been observed during aging and many chronic diseases [56,57]. Thus, mitochondrial damage results not only in the loss of energy production but also exposes cells to oxidative damage. In the presented study we have observed decreased mitochondrial membrane potential in OCD chondrocytes and decreased expression of mitophagy promoting PINK. Mitophagy leads to the removal of depolarized and damaged mitochondria but in OCD chondrocytes that mechanism seems to be inhibited. Ansari et al. [19] suggested that mitophagy is a crucial mechanism which prevents ROS production and enhances the survival, however, of OA chondrocytes and that loss of mitophagy directly contributes to development of the disease. Accumulation of damaged mitochondria leads to ROS overload and, in consequence, to chondrocyte apoptosis. Compared with healthy cartilage, deteriorated chondrocytes fail to generate energy due to alternation in mitochondrial dynamics. In our study we have revealed that mitochondria from OCD chondrocytes are characterized by increased fusion which allows for the exchange of content including DNA, and metabolites between neighboring organelles, including damaged or senescent ones, in consequence promoting their survival [58]. Thus we speculate, that in the state of decreased mitophagy, mitochondrial fusion serves as a compensatory mechanism which mitigates the effects of mitochondria damage through the exchange of proteins and lipids with other organelles.

Autophagy is a process that has gained increased attention in OA development as it was shown to be triggered in hypertrophic chondrocytes [59]. However, autophagy has two faces and can serve as pro-apoptotic or a protective mechanism which is lost during OA [10]. However, the exact role of autophagy in OCD development since now has not been studied. Chang et al. have shown that the induction of autophagy prevented the accumulation of subdiploid cells in young chondrocytes, while it induced cell death by autophagy in OA chondrocytes [60]. Moreover, they did not observe differences in the expression of LC-3 and Beclin between healthy and OA chondrocytes. This is in good agreement with our data as we did not observe macroautophagy flux in OCD cells. However, we observed up-regulation of genes involved with the chaperone mediated autophagy which removes damaged proteins by directing them into lysosomes. Thus we speculate that that CMA may play a protective role in OCD chondrocytes. We speculate that CMA activates before macroautophagy as a last chance rescue mechanism as it was shown that advanced OA contributes to “chondropoptosis” in different layers of the articular cartilage [61].

Moreover, we have observed that LPS treated OCD cells while co-cultured with ASC displayed decreased expression of SQSTM and FIS while increased expression of MMP-13. This is an interesting phenomenon which indicates that during co-culture with ASC, mitochondrial fission and their removal through mitophagy may be diminished. On the contrary, Jiang et al. [62] have demonstrated that ASCs activate autophagy and inhibit the expression of MMPs in chondrocytes treated with IL-1β or LPS. Interestingly, we have observed significantly increased expression of MM-13 in LPS treated OCD chondrocytes. This may indicate that stem cells should not be applied to tremendously inflamed joints as they may exert the opposite effects. However, this phenomenon should be further investigated.

## 5. Conclusions

The presented study provides insights into the biology of horse OCD chondrocytes and the involvement of oxidative stress, mitochondria damage, and autophagy in the pathogenesis of disease from the point of chondrocytes’ senescence. Our findings show on the decreased antioxidative capacities and increased NO levels in OCD chondrocytes and resulting mitochondria damage followed by cellular dysfunction. Furthermore, our results indicate that deterioration of those cells is also correlated with ER stress. We postulate that in order to deal with the accumulation of misfolded proteins and their aggregates, OCD chondrocytes activate chaperone-mediated autophagy as a rescue mechanism to remove harmful biomolecules and are damaged by ROS organelles. New efforts to prevent the development and progression of OCD in horses may include strategies aimed at the efficient protection and improvement of mitochondrial activity by reducing ROS levels and chondrocyte apoptosis would be a valid target to modulate cartilage degeneration. OCD chondrocytes share similar characteristics with OA chondrocytes, which indicates that the molecular basis of these diseases’ pathophysiology are common.

## Figures and Tables

**Table 1:** Sequences of primers used in reverse transcription polymerase chain reaction (RT-PCR). ACAN—aggrecan; DCN—decorin; COMP—cartilage oligomeric matrix protein; MMP-13—matrix metallopeptidase 13; p53—p53 tumor suppressor protein; p21—cyclin-dependent kinase inhibitor 1; Cas-3—caspase 3; Cas-9—caspase 9; CHOP—C/EBP homologous protein; PERK—Eukaryotic translation initiation factor 2-alpha kinase 3; eIF2-alpha—eukaryotic initiation factor 2 alpha; BECLIN—beclin-1; LAMP-2—lysosome-associated membrane protein 2; SQSTM—p62/sequestosome 1; LC-3—microtubule-associated protein 1A/1B-light chain 3; FIS—mitochondrial fission 1 protein; PINK—PTEN-induced kinase 1; PARKIN—parkin ligase; MNF—mitofusin 1; Coll-9—collagen IX; RUNX-2—runt-related transcription factor 2; RUNX-3—runt-related transcription factor 3; MIEF-1—mitochondrial elongation factor 1; MIEF-2—mitochondrial elongation factor 2; HSC70—heat shock cognate 71 kDa protein; BIP—binding immunoglobulin protein; GAPDH—glyceraldehyde 3-phosphate dehydrogenase.

Gene Name	Forward and Reverse Primer Sequence (5′-3′)	Size of Amplicon (bp)	Accession No.
*ACAN*	F: TGGTGTCCTCTTCTTGTCGCTTTC	160	XM_014733894.1
	R: ACGATACATTTGCTGTGCTTCGGC		
*DCN*	F: GATGCAGCTAGCCTGAGAGG	248	XM 014841263.1
	R: GTGTTGTATCCAGGTGGGCA		
*COMP*	F: AGTGTCGCAAGGATAACTGCGTGA	238	NM_001081856.1
	R: TCCTGATCTGTGTCCTTCTGGTCA		
*MMP-13*	F: TGCAGCTGTTCACTTTGAGGA	200	NM_001081804.1
	R: ACTGTATGGGCCCGTTGAAA		
*p53*	F: TACTCCCCTGCCCTCAACAA	252	U37120.1
	R: AGGAATCAGGGCCTTGAGGA		
*p21*	F: GAAGAGAAACCCCCAGCTCC	241	XM_014853747.1
	R: TGACTGCATCAAACCCCACA		
*Cas-3*	F: GGCAGACTTCCTGTATGCGT	167	XM_023630401.1
	R: CCATGGCTACCTTGCGGTTA		
*Cas-9*	F: CACCTTCCCAGGCTTTGTCT	224	XM_005607504.3
	R: GGCTCTGGCCTCAGTAAGTT		
*CHOP*	F: AGCCAAAATCAGAGCCGGAA	272	XM 014844003.1
	R: GGGGTCAAGAGTGGTGAAGG		
*PERK*	F: GTGACTGCAATGGACCAGGA	283	XM 014852775.1
	R: TCACGTGCTCACGAGGATATT		
*eIF2-alpha*	F: AGTCTTCAGGCATTGGCTCC	489	XM_001488848.6
	R: CCGAGTGGGACATGTATCGG		
*BECLIN*	F: GATGCGTTATGCCCAGATGC	233	XM 014833759.1
	R: AACGGCAGCTCCTCTGAAAT		
*LAMP-2*	F: GCACCCCTGGGAAGTTCTTA	139	XM 014733098.1
	R: ATCCAGCGAACACTCTTGGG		
*SQSTM*	F: CATCGGAGGATCCCAGTGTG	207	XM_005599173.3
	R: CCGGTTTGTTAGGGTCGGAA		
*LC-3*	F: TTCTGAGACACAGTCGGAGC	128	XM_001493613.6
	R: CTTTGTTCGAAGGTGTGGCG		
*FIS*	F: GGTGCGAAGCAAGTACAACG	118	XM 001504462.4
	R: GTTGCCCACAGCCAGATAGA		
*PINK*	F: GCACAATGAGCCAGGAGCTA	298	XM 014737247.1
	R: GGGGTATTCACGCGAAGGTA-0		
*PARKIN*	F: TCCCAGTGGAGGTCGATTCT	218	XM 014858374.1
	R: CCCTCCAGGTGTGTTCGTTT		
*MNF*	F: AAGTGGCATTTTTCGGCAGG	217	XM 001495170.5
	R: TCCATATGAAGGGCATGGGC		
*Coll-9*	F: CGGTGAAAAGGGCAACACAG	260	XM_014734623.1
	R: GTTCACCGACTACACCCCTG		
*RUNX-2*	F: ACTTTGCAGAGATGGGCCTC	76	XM_005603968.2
	R: CTAGGAAGTCGGGATGGGGA		
*RUNX-3*	F: ATCAAGGTGACCGTGGATGG	219	XM_008532790.1
	R: GGAGAAAGGGTTCAGGTCCG		
*MIEF-1*	F: ATGCTGGGCATCGCTACAC	284	XM_023631522.1
	R: CGGAGCCGTGACTTCTTCAA		
*MIEF-2*	F: CGTTCTATTATCAGGCAGGTCC	108	XM_005597824.3
	R: AGAACTCTGCCATGGTCTTCT		
*HSC70*	F: GATTAACAAGAGGGCTGTCCGTC	122	XM_023628864.1
	R: GCCTGGGTGCTAGAAGAGAGA		
*BIP*	F: CTGTAGCGTATGGTGCTGCT	122	XM_005606029.2
	R: CATGACACCTCCCACGGTTT		
*GAPDH*	F: GATGCCCCAATGTTTGTGA	250	NM 001163856.1
	R: AAGCAGGGATGATGTTCTGG		
